# Maternal Overweight Downregulates MME (Neprilysin) in Feto-Placental Endothelial Cells and in Cord Blood

**DOI:** 10.3390/ijms21030834

**Published:** 2020-01-28

**Authors:** Elisa Weiß, Hannah M. Berger, Waltraud T. Brandl, Jasmin Strutz, Birgit Hirschmugl, Violeta Simovic, Carmen Tam-Ammersdorfer, Silvija Cvitic, Ursula Hiden

**Affiliations:** 1 Department of Obstetrics and Gynecology, Medical University of Graz, 8036 Graz, Austria; 2 Otto Loewi Research Center for Vascular Biology, Immunology and Inflammation, Medical University of Graz, 8036 Graz, Austria

**Keywords:** MME, neprilysin, maternal overweight, feto-placental endothelial cells, umbilical cord blood

## Abstract

Maternal overweight in pregnancy alters the metabolic environment and generates chronic low-grade inflammation. This affects fetal development and programs the offspring’s health for developing cardiovascular and metabolic disease later in life. MME (membrane-metalloendopeptidase, neprilysin) cleaves various peptides regulating vascular tone. Endothelial cells express membrane-bound and soluble MME. In adults, the metabolic environment of overweight and obesity upregulates endothelial and circulating MME. We here hypothesized that maternal overweight increases MME in the feto-placental endothelium. We used primary feto-placental endothelial cells (fpEC) isolated from placentas after normal vs. overweight pregnancies and determined MME mRNA, protein, and release. Additionally, soluble cord blood MME was analyzed. The effect of oxygen and tumor necrosis factor α (TNFα) on MME protein in fpEC was investigated in vitro. Maternal overweight reduced MME mRNA (−39.9%, *p* < 0.05), protein (−42.5%, *p* = 0.02), and MME release from fpEC (−64.7%, *p* = 0.02). Both cellular and released MME protein negatively correlated with maternal pre-pregnancy BMI. Similarly, cord blood MME was negatively associated with pre-pregnancy BMI (*r* = −0.42, *p* = 0.02). However, hypoxia and TNFα, potential negative regulators of MME expression, did not affect MME protein. Reduction of MME protein in fpEC and in cord blood may alter the balance of vasoactive peptides. Our study highlights the fetal susceptibility to maternal metabolism and inflammatory state.

## 1. Introduction

Pregnancies complicated by maternal overweight are associated with an altered metabolic and endocrine environment and characterized by chronic low-grade inflammation [1,2]. These changes affect fetal development and, furthermore, program the offspring’s health in the long term (reviewed by Ingvorsen et al. [3] and Zhou and Pan [4]). For instance, higher maternal pre-pregnancy BMI is associated with an increased childhood blood pressure [5,6] and an altered childhood cardiac structure [7]. Moreover, maternal overweight in pregnancy is positively associated with the development of cardiovascular disease in adult offspring [8]. Thus, although the mechanisms are not fully understood, there is a well-established association of maternal overweight and obesity with endothelial function and cardiovascular disease. Mechanisms may involve both direct mechanical effects of altered fetal hemodynamics on fetal cardiac development, as well as programming effects of the changed intrauterine environment on endothelial cells, cardiomyocytes, and smooth muscle cells.

MME (membrane metalloendopeptidase, also termed neprilysin and CD10) is an integral membrane-bound zinc metallo-endopeptidase that cleaves various vasoactive peptides and thus participates in blood pressure regulation [9]. Specifically, MME catalyzes degradation of several vasodilator peptides, including ANP, BNP, and CNP (natriuretic peptides A, B and C), adrenomodulin and bradykinin, as well as vasoconstrictor peptides such as endothelin-1 and angiotensin II (reviewed by Corti et al. [10]). Thus, the overall effect of MME on vascular tone regulation depends on the presence and composition of substrates within the specific vascular system [10].

MME is expressed on various cell types including endothelial cells [9]. Besides the membrane-bound form, a soluble form of MME exists that retains catalytic activity [11]. In fact, endothelial cells are a source of soluble MME [12]. Under metabolic derangements, soluble MME increases in the systemic circulation in adults, and correlates positively with BMI [13,14]. Various factors of the altered metabolic and pro-inflammatory environment contribute to this upregulation—in vitro experiments revealed that hyperglycemia and hyperlipidemia increase MME production in endothelial cells [15]. Additionally, the correlation of circulating MME levels with CRP (C-reactive peptide) reveals the susceptibility of MME to the pro-inflammatory environment, and pro-inflammatory mediators such as IL-β1 (interleukin β1) and TGFβ (transforming growth factor β) increase MME in various cell types [16,17,18].

We here hypothesized that maternal overweight upregulates MME in the fetal endothelium. To this end, we isolated primary human feto-placental endothelial cells after pregnancies of normal weight and overweight women and measured MME mRNA and protein.

## 2. Results

### 2.1. Feto-Placental Endothelial Cells (fpEC) Expressed MME mRNA and Protein In Vivo and In Vitro

**Figure 1:**
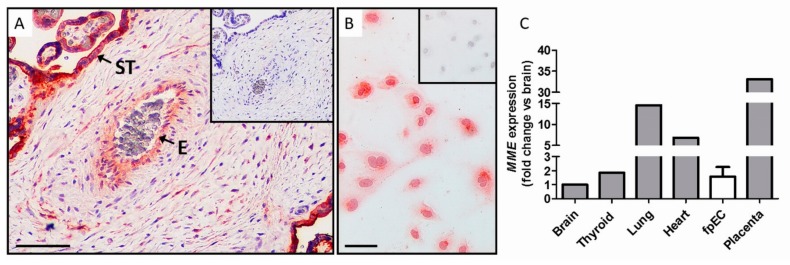
MME (membrane metalloendopeptidase) protein and mRNA expression in feto-placental endothelium. (**A**) In placental tissue, positive staining for MME (red) was detected in the syncytiotrophoblast (ST) facing the maternal circulation, as well as in the feto-placental endothelium (E) facing the fetal circulation. Nuclei were stained blue with DAPI (4′,6-diamidino-2-phenylindole). Scale bar: 100 µm. (**B**) Immunocytochemistry revealed that isolated primary feto-placental endothelial cells (fpEC) continued to express MME in culture. Scale bar: 200 µm. Negative controls using unspecific mouse IgG are shown in the inserts. (**C**) Comparison of *MME* mRNA expression in different classical MME-producing tissues and organs, and in fpEC and placenta. Data were normalized to the mean of the house-keeping genes hypoxanthine-guanine phosphoribosyltransferase (*HPRT1*) and peptidylprolyl isomerase A (*PPIA*) and represented in relation to the expression in brain.

To confirm MME protein production in placental endothelium, we performed immunohistochemistry of placental tissue. In fact, there was a positive staining for MME protein in the endothelium of the feto-placental vessels (Figure 1A). Furthermore, there was an intense MME staining also in the syncytiotrophoblast, the classical placental barrier that contacts the maternal bloodstream. Immunocytochemistry of isolated primary fpEC also further revealed production of MME in cultured fpEC (Figure 1B). Quantitative RT-PCR was used to compare *MME* expression levels of primary fpEC to *MME* expression in various classical MME-producing human organs (Figure 1C) and revealed feto-placental *MME* levels comparable to brain and thyroid. Total placental tissue revealed the highest expression of all analyzed organs, due to the intense MME expression in the syncytiotrophoblast.

### 2.2. Maternal Pre-Pregnancy Overweight Reduced MME mRNA and Protein in fpEC

**Figure 2:**
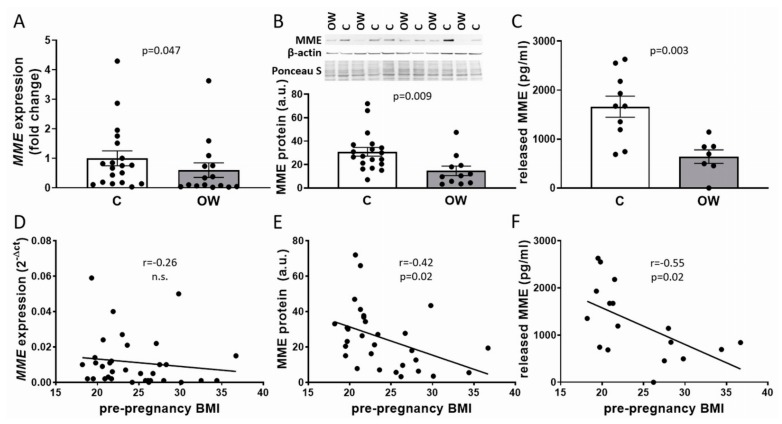
*MME* mRNA and protein in fpEC after normal and overweight pregnancy. *MME* mRNA (**A**), cellular protein (**B**), and released MME (**C**) was reduced in primary fpEC exposed to maternal overweight (*t*-test). When correlated to maternal pre-pregnancy BMI (Pearson correlation), this association was not significant for fpEC *MME* mRNA (**D**), but it was significant for fpEC protein production (**E**) and release (**F**). *MME* mRNA was normalized to the mean of the housekeeping genes *HPRT1* and ribosomal protein L30 (*RPL30*), respectively. A representative immunoblot for MME, β-actin, and the Ponceau S staining of the corresponding membrane are shown on top of the protein data in (B). C: controls; OW: overweight; a.u.: arbitrary units. *MME* mRNA: *n*(c) = 19; *n*(ow) = 15; cellular MME: *n*(c) = 19; *n*(ow) = 11; MME release: *n*(c) = 10; *n*(ow) = 7. The bars in (**A**–**C**) represent the mean ± SEM.

Analysis of *MME* mRNA expression in primary fpEC isolated after pregnancies of women with normal vs. overweight BMI (Table 1) revealed a reduction of *MME* in fpEC exposed to overweight pregnancies (−39.9%, *p* = 0.047) (Figure 2A). This was paralleled by a reduction of cellular MME protein (−42.5%, *p* = 0.02) as well as secreted MME in the culture medium (−64.7%, *p* = 0.02) (Figure 2B,C). Whilst there was no significant correlation between fpEC *MME* mRNA expression and maternal pre-pregnancy BMI, cellular MME protein and MME secretion negatively correlated with BMI (*r* = −0.42, *p* = 0.02 and *r* = −0.55, *p* = 0.02, respectively) (Figure 2D–F).

### 2.3. Maternal Pre-Pregnancy Overweight Reduced Umbilical Cord Blood MME Levels

**Figure 3:**
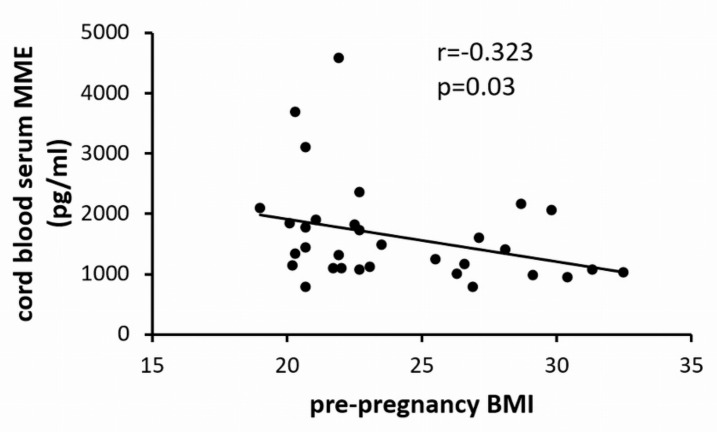
Correlation of umbilical cord blood serum MME levels with maternal pre-pregnancy BMI (*n* = 32).

Exposure to the intrauterine environment of overweight reduced MME release by fpEC in vitro. This raised the question as to whether maternal overweight also alters soluble MME in the fetal circulation. Thus, we collected a cohort of umbilical cord blood sera of pregnancies with normal vs. overweight pre-pregnancy BMI (Table 2). In parallel to the findings in isolated primary fpEC, MME in cord blood serum correlated negatively with maternal pre-pregnancy BMI (Figure 3).

**Figure 4:**
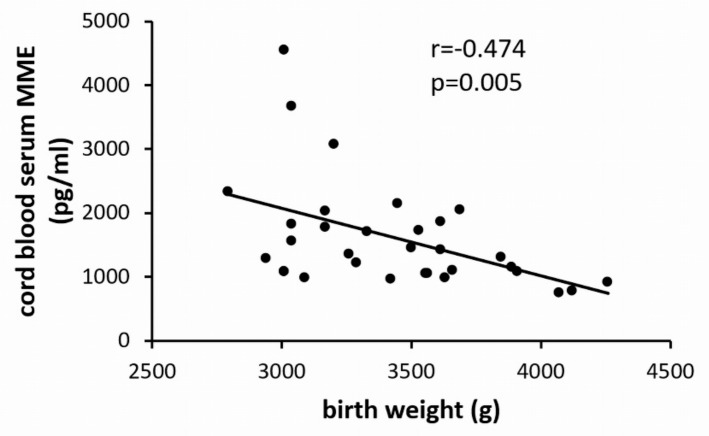
Correlation of umbilical cord blood serum MME levels with birth weight (*n* = 32).

This opposes findings in adults demonstrating upregulation of circulating MME with increasing BMI [13,14]. We therefore investigated whether the reduction of MME in cord blood serum is determined not by maternal BMI, but by fetal weight. However, similar to maternal BMI, neonatal weight also correlated negatively with cord blood MME (Figure 4).

### 2.4. MME Protein in fpEC was not Regulated by Oxygen and Tumor Necrosis Factor α (TNFα)

**Figure 5:**
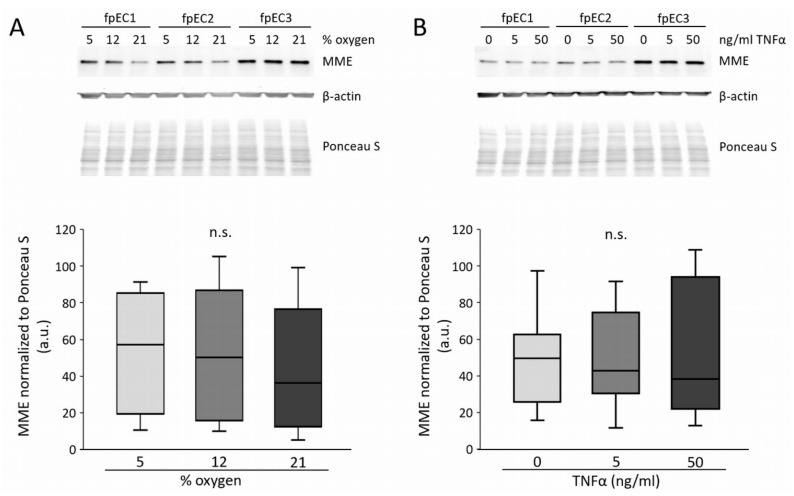
Effect of oxygen and tumor necrosis factor α (TNFα) on MME protein in fpEC. (**A**) MME protein after culture at 5%, 12%, and 21% oxygen for 48 h. (**B**) MME protein after TNFα treatment (0, 5, and 50 ng/mL) for 24 h. Protein levels of β-actin were used as loading controls and protein was normalized to total protein staining with Ponceau S. Experiments were performed in *n* = 7 different fpEC isolations. Representative immunoblots of three fpEC isolations are shown on top. a.u.: arbitrary units; *n*.s.: not significant.

Hypoxia and NF-κB (nuclear factor kappa B) signalling downregulate MME in other cell types [19,20,21], and TNFα (tumor necrosis factor α) is an activator of NF-κB signaling [22]. Thus, we tested whether oxygen or TNFα altered MME in fpEC from control pregnancies. However, after 48 h, MME protein did not differ between cells grown at 5%, 12%, and 21% oxygen (Figure 5A). Additionally, TNFα treatment (5 and 50 ng/mL) did not affect MME after 24 h (Figure 5B).

## 3. Discussion

Obesity and metabolic syndrome in adults are associated with increased levels of MME [13,14], and this may modulate vascular tone regulation. Upregulation of MME by fatty acids, glucose, and the pro-inflammatory environment represent a possible underlying reason [15,16,17]. In utero exposure to maternal overweight primes the offspring to a disturbed vascular tone regulation in childhood with elevated blood pressure and an adverse cardio-metabolic risk profile [3,4,5,6,7,8]. This study tested the hypothesis that the moderate metabolic derangement of maternal overweight in pregnancy increases the expression and release of MME by primary fpEC isolated from the feto-placental vasculature.

Surprisingly, maternal overweight reduced MME levels in endothelial cells and in the fetal circulation. In addition, fetal weight was negatively correlated with circulating cord blood serum MME. Despite normal maternal oGTT (oral glucose tolerance test) levels, maternal overweight and obesity in pregnancy are characterized by insulin resistance and increased levels of inflammatory markers, i.e., IL-6 [23] and CRP [24]. Additionally, in our overweight samples revealing only moderate metabolic derangement with normal oGTT, maternal CRP was increased (Table 2) and positively correlated with maternal pre-pregnancy BMI (Supplementary Figure S1). This altered maternal metabolic and pro-inflammatory environment translates also to the fetal circulation, as fetal insulin resistance, increased cord blood TNFα, and CRP arise [25,26,27,28]. However, whilst insulin resistance and inflammatory status are associated with elevated MME in adults [16,17,18], other mechanisms seem to account for MME regulation in the fetal compartment.

Possible insults downregulating MME include hypoxia and NF-κB (nuclear factor kappa B) signaling. In rodents, hypoxia downregulates MME expression in the lungs, kidneys [20], and in the brain [21]. In fact, several studies have revealed a subtle hypoxic fetal environment in pregnancy complicated by maternal overweight [29,30]. Additionally, MME is downregulated by NF-κB pathway via microRNA miR155 in B-lymphoma cells [31]. The pro-inflammatory cytokine TNFα activates NF-κB pathway [22], which subsequently upregulates ICAM-1 (intercellular adhesion molecule 1) expression [32]. TNFα is increased in cord blood when mothers are overweight [27], and fpEC also respond to TNFα with an induction of ICAM-1 expression [33]. Thus, we further tested whether hypoxia or TNFα reduce MME protein in fpEC in vitro. However, both conditions did not affect MME protein, suggesting that neither hypoxia nor NF-κB participate in the overweight-associated reduction of MME. Fitzpatrick et al. [34] discovered that shear stress downregulates MME in aortic endothelial cells by a mechanism involving NADPH oxidase-dependent production of reactive oxygen species (ROS). Nonetheless, the unaffected expression of MME in fpEC exposed to different oxygen concentrations does not point towards a ROS-dependent signaling event accounting for MME reduction in overweight pregnancies. Besides signaling events, various studies have revealed epigenetic mechanisms underlying changes in fetal gene expression in response to an altered intrauterine environment. Such epigenetic mechanisms include DNA methylation and translational repression by miRNAs (reviewed by [35]). In fact, both mechanisms occur in MME regulation: Increased DNA methylation of the *MME* promoter region was observed in Alzheimer’s disease brain [36] and in several types of cancer, such as leukemia [37] and breast cancer [38]. Additionally, MME is a target of miRNA-mediated regulation, as shown for the abovementioned miR155 [31]. Moreover, the miRNA target prediction database miRDB (www.mirdb.org) revealed that 156 different miRNAs potentially target *MME* mRNA, and thus may participate in its translational repression.

The physiological outcome of reduced endothelial and circulating MME can only be hypothesized and may be—in parallel with MME’s multifunctional action—versatile. Reduced MME levels may affect the balance of vasoactive peptides, therefore affecting vascular tone regulation [10]. In fact, in human umbilical vein endothelium, MME effectively inactivates bradykinin, and hence impairs bradykinin-mediated vasodilatation [39]. However, depending on the presence and composition of MME vasoactive substrates within a certain vascular bed, and depending on the phenotype and responsiveness of the specific endothelial cells to these substrates [10], MME effects may differ in the fetus or neonate.

Moreover, recent literature suggests that elevated MME participates in the development of insulin resistance. On one hand, MME cleaves peptides stimulating insulin secretion, i.e., glucagon [40] and GLP1 (glucagon-like peptide 1) [41], and thus MME may reduce systemic insulin secretion [42]. On the other hand, membrane-associated MME modulates internalization and cellular localization of the insulin receptor, as shown by *MME* knockdown and overexpression in insulin target cells [43]. Hence, decreased levels of MME, as we observed in the overweight cohort, may play a role in the deregulation of insulin response and insulin resistance. In mice *MME*, knockout results in increased insulin sensitivity [43]. However, neonates from pregnancies complicated by maternal obesity have increased insulin resistance [24]. Thus, the role and specific effect of reduced MME in feto-placental endothelium and cord blood on insulin secretion and resistance is difficult to predict.

Besides the regulation of vascular tone and insulin sensitivity, a role of MME in the regulation of angiogenesis was suggested. On one hand, MME cleaves and inactivates pro-angiogenic [44,45] as well as anti-angiogenic [46,47] acting growth factors and peptides. On the other hand, MME located at the cell membrane seems to participate in signaling events and has been shown to prevent FAK (focal adhesion kinase) activation and attenuates PKB (protein kinase B) signaling, causing reduced migration and angiogenesis (reviewed by Maguer-Satta et al. [48]). Several reports describe distinct placental vascular structure as a result of obesity in human and animals, with either increased [49,50,51] or decreased [52,53,54] vascularity, suggesting that placental angiogenesis and vascular development is susceptible towards maternal metabolic and pro-inflammatory changes, but the effective result may depend on the specific situation, i.e., moderate vs. severe metabolic changes. In any event, altered angiogenesis in the placenta will generate distinct placental vascular structure and architecture, ultimately affecting hemodynamics. This may contribute to functional programming of the fetal cardiovascular system.

We see it as a limitation of our study that we do not have further metabolic and inflammatory information on the subjects, both from the maternal and from the fetal side. Data on maternal insulin resistance, fetal C-peptide, erythropoietin, and inflammatory markers would add significantly to the knowledge about the specific intrauterine stimulus downregulating MME and should be included in further studies.

Our study demonstrates for the first time that the moderate metabolic derangements of maternal overweight decreases feto-placental endothelial and fetal circulating MME, thus highlighting the susceptibility of the fetus to maternal metabolism and low-grade inflammation.

## 4. Materials and Methods

### 4.1. Sample Collection

Ethical approval was obtained from the Medical University of Graz (approval reference number 29–319 ex 16/17, 29.06.2017) and all women provided written informed consent. Placentas for feto-placental endothelial cell (fpEC) isolation were collected from pregnancies of non-smoking (self-reported) women with a negative 75 g oral glucose tolerance test (oGTT) performed at 25–28 weeks of gestation, free from any medical disorders or pregnancy complications. Control placentas were obtained from women with a pre-pregnancy BMI < 25, overweight placentas were collected from women with a pre-pregnancy BMI > 25. Table 1 shows the characteristics of the fpEC donors.

For the collection of umbilical cord blood serum, the same inclusion and exclusion criteria were used. Umbilical cord blood was collected directly after delivery, centrifuged at 3000 rpm for 10 min at 4 °C, and the serum was then stored at −80 °C. Table 2 shows the characteristics of the cord blood donors.

### 4.2. Cell Culture

Primary arterial feto-placental endothelial cells (fpEC) were isolated from the collected placentas following a standard protocol [55]. Briefly, chorionic arteries were dissected and endothelial cells isolated by perfusion with a collagenase/dispase (Roche, Mannheim, Germany) solution. Cells were resuspended in endothelial basal medium (EBM, Lonza, Walkersville, MD, USA) supplemented with the EGM-MV BulletKit (Lonza) on 1% (*v*/*v*) gelatin-coated flasks. Isolated fpECs were grown at 37 °C and 12% oxygen, and used up to passage 10. Cells were characterized by immunocytochemical analysis (c.f. below) with positive staining for the endothelial cell markers VWF (von Willebrand factor) and CD31, and negative for the SMA (smooth muscle actin) and the fibroblast marker CD90. For isolation of RNA, protein, and collection of supernatant, cells were grown in 75 cm² flasks to approximately 90% confluency, washed with ice-cold Hank’s balanced salt solution (HBSS; Gibco, Thermo Fisher Scientific, Runcorn, United Kingdom), and harvested as described below.

### 4.3. Immunohistochemistry

Immunohistochemical staining of MME was performed on standard formalin-fixed paraffin embedded term placenta sections (5 µm). Standard deparaffinization procedure was followed by boiling slides in Epitope Retrieval Solution pH 9.0 (Novocostra, Leica, Vienna, Austria) for 7 min at 120 °C in a decloaking chamber (Biocare Medical, Pacheco, CA, USA). Sections were immunostained using the UltraVision horseradish peroxidase (HRP) Polymer Kit (Thermo Fisher Scientific) according to the manufacturer’s protocol. Briefly, endogenous peroxidase was blocked using the hydrogen peroxidase block for 10 min. Three washing steps with Tris-buffered saline (TBS) were followed by background blocking using Ultra Vision Protein Block for 5 min. Monoclonal mouse anti-CD10 antibody (Thermo Fisher Scientific) was diluted at 1:2000 in Antibody Diluent (Dako, Glostrup, Denmark) and incubated on slides for 45 min at RT (room temperature). Slides were washed and detection achieved by incubation with the anti-mouse/rabbit UltraVision HRP-labelled polymer system (15 min) and 3-amino-9-ethylcarbacole (AEC, Thermo Fisher Scientific), according to the manufacturer’s instructions. Nuclei were stained with Haemalaun solution (Sigma, St. Lois, MO, USA) and slides were mounted with aqueous mounting agent Aquatex (Merk Millipore, Darmstadt, Germany). For negative controls, slides were incubated with the same concentration of unspecific mouse IgG1 (Dako) as the primary antibody. Images were acquired using a Zeiss Axiophot microscope equipped with an AxioCamHRc digital camera.

### 4.4. Immunocytochemistry

Immunocytochemistry for quality control of fpEC and for MME was performed according to the same protocol. Cells (100,000 cells per 1.7 cm^2^ chamber) were grown on chamber slides for 48 h, washed with HBSS, and fixed with ice-cold acetone (Merck, Darmstadt, Germany) for 3 min. Slides were rehydrated in Tris-borate EDTA (TBE) pH 7.5 with 0.1% Tween (Sigma) for 3 min, which was also used as a washing puffer. Non-specific binding sites were blocked with UltraVision Protein Block for 10 min. Subsequently, the primary antibody for endothelial cell markers VWF (anti-VWF A0082, Dako; 1:3000) and CD31 (anti-CD31 MON6002-1, clone EN4, Monosan, Uden, the Netherlands; 1:300), smooth muscle cell marker SMA (anti-SMA M0851, clone 1A4, Dako, 1:200), fibroblast marker CD90 (anti-CD90, DIA100, clone AS02, Dianova, Hamburg, Germany; 1:200), and MME (anti-CD10; Thermo Fisher Scientific; 1:100) diluted in Dako antibody diluent was applied for 30 min. Negative controls were of the same isotype and in the same dilution (Dako). After three washings, slides were incubated with HRP polymer for 15 min in the dark and washed again. Then, chromogenic reaction was started by addition of peroxidase-compatible chromogen (Thermo Fisher Scientific) for 5 min. After washing in distilled water, nuclei were stained with hematoxylin and mounted with Aquatex.

### 4.5. Quantitative Reverse Transcription PCR (RT-qPCR)

Total RNA was isolated using the miRNeasy mini kit (Qiagen, Hilden, Germany). The quality and integrity of the RNA was determined by the ratio of spectrophotometric absorbance 260 nm/280 nm measured with the Scandrop 250 (Analytik Jena AG, Jena, Germany). Complementary DNA was transcribed from 1 µg of total RNA using miScript II RT kit and 5× miScript HiFlex Buffer (Qiagen) according to the manufacturer’s instructions.

For the quantification of *MME* mRNA in primary fpEC, 3 ng/µL of cDNA was used per reaction in a total reaction volume of 20 µL in a CFX96 cycler (BioRad, Hercules, CA, USA). RT-qPCR for *MME* was performed using the TaqMan assay Hs00155310_m1 (Applied Biosystems, CA, USA) for *MME*, and Hs02800695_m1 and Hs00265497_m1 (hypoxanthine-guanine phosphoribosyltransferase (*HPRT1*) and ribosomal protein L30 (*RPL30*), respectively) were used as housekeeping genes. For the quantification of *MME* mRNA in different human organs, 40 ng of cDNA was used, which was transcribed from fpEC and purchased RNA from human tissues (Clonetech; Thermo Fisher Scientific). For the comparison of gene expression between different organs, the housekeeping genes *HPRT1* (Hs02800695_m1) and peptidylprolyl isomerase A (*PPIA,* Hs04194521_s1) were used [56]. Mean expression of the housekeeping genes was used to normalize gene expression with 2^−∆∆*C*t^ method.

### 4.6. Immunoblot

Total cellular protein was extracted with RIPA buffer containing proteinase inhibitors (Complete Protease Inhibitor Cocktail Tablets, Roche). Cell lysates (8 µg per lane) were applied to a gradient 4–20% SDS-PAGE, and transferred to 0.2 µm nitrocellulose membranes (Trans-Blot Turbo Mini Nitrocellulose Transfer Membrane, BioRad) using the Trans-Blot Turbo Transfer System (BioRad). After transfer, membranes were incubated with Ponceau S solution (Sigma), which stained all transferred proteins. The membranes were photographed for protein normalization later. For detection of MME, mouse monoclonal anti-CD10 antibody (clone SN5c, Abcam, CA, USA) was used at a 1:1000 dilution. The secondary antibody was HRP-conjugated goat anti-mouse antibody (1:1000; R&D Systems). Signals were detected using the SuperSignal West Pico (Pierce, Thermo Fisher Scientific). For control of antibody detection, membranes were incubated with mouse monoclonal anti-β-actin (1:20,000; clone AC-15, Abcam) followed by incubation with the HRP-conjugated secondary antibody (1:20,000). MME signals were detected at 85 kDa and normalized to the Ponceau S-stained proteins in the molecular range between 40 and 100 kDa. Normalization of signals was performed with the signal intensity and was calculated by DigiDoc 1000 software.

### 4.7. Enzyme-Linked Immunosorbent Assay (ELISA)

For the collection of supernatants, fpEC were seeded (15,000 cells/cm^2^) in T25 flasks and cultured for 48 h. Then, supernatants were centrifuged at 3000 rpm for 10 min and frozen at −80 °C until the experiment. Absorbance of medium without cells incubated the same way was substracted from MME levels measured in the conditioned medium. Cord blood serum was obtained as described above. The human Neprilysin (MME) ELISA kit (Abnova, Taoyvan City, Taiwan) was performed according to the manufacturer’s instructions. A total of 100 µL of cell culture supernatants diluted at 1:4 and 100 µL of cord blood serum were applied. Optical density was determined at 450 nm using a spectrophotometer (SPECTRO Analytical Instruments, Kleve, Germany).

### 4.8. Hypoxia and TNFα Treatments

To test the effect of hypoxia or TNFα on MME protein expression, fpEC (*n* = 7 different cell isolations, in duplicates) were seeded in gelatin coated 6-well plates (200,000 cells/well). Then, culture plates were placed at different oxygen concentrations (21%, 12%, and 5%) for 48 h. For TNFα treatment, after overnight culture, cells were treated with TNFα (5 and 50 ng/mL, Reliatech, Wolfenbüttel, Germany) for 24 h. Cells without TNFα treatment served as the control. Protein was extracted with RIPA buffer containing proteinase inhibitors (complete), and immunoblotting for MME was performed as described above.

### 4.9. Statistical Analysis

Data were analyzed using GraphPad Prism software Version 5.01 (GraphPad Software, Inc). After testing for normal distribution (Kolmogorov–Smirnov test), Student’s *t*-test and Pearson’s test were applied to detect differences between the control and overweight samples and correlations, respectively. *p*-values below 0.05 were considered statistically significant.

## Figures and Tables

**Table 1:** Characteristics of the fpEC donors.

Characteristics	Controls	Overweight Subjects
Number of cases	19	15
Pre-pregnancy BMI (kg/m^2^)	21.1 ± 1.7	28.7 ± 3.4 ***
BMI at birth (kg/m^2^)	26.9 ± 2.4	33.9 ± 2.6 ***
Maternal age (years)	34.0 ± 5.7	32.3 ± 4.1
oGTT (0 h)	80.6 ± 5.9	82.9 ± 4.7
oGTT (1 h)	105.9 ± 27.8	116.5 ± 27.8
oGTT (2 h)	95.6 ± 20.5	94.1 ± 16.3
Gestational age at delivery (weeks)	39.7 ± 1.0	39.2 ±1.6
Mode of delivery (vaginal/C-section)	8/11	6/9
Fetal weight (g)	3559 ± 351	3477 ± 402
Fetal height (cm)	51.5 ± 1.7	51.3 ± 2.5
Fetal sex (m/f)	12/7	8/7
Placental weight (g)	634 ± 117	602 ± 147

*** indicates *p* < 0.001. oGTT: oral glucose tolerance test.

**Table 2:** Characteristics of the cord blood donors.

Characteristics	Controls	Overweight Subjects
Number of cases	20	12
Pre-pregnancy BMI (kg/m^2^)	21.4 ± 1.2	28.6 ± 2.4 ***
BMI at birth (kg/m^2^)	26.7 ± 2.1	33.1 ± 2.2 ***
Maternal age (years)	31.1 ± 5.3	27.8 ± 3.1
oGTT (0 h)	81.1 ± 5.7	83.6 ± 4.8
oGTT (1 h)	123.5 ± 34.7	112.2 ± 20.4
oGTT (2 h)	98.8 ± 24.4	102.4 ± 20.5
Maternal CRP at delivery	2.8 ± 1.9	4.9 ± 2.6 *
Gestational age at delivery (weeks)	39.1 ± 0.9	38.9 ± 0.9
Mode of delivery (vaginal/C-section)	3/17	1/11
Fetal weight (g)	3358 ± 370	3493 ± 385
fetal height (cm)	50.5 ± 2.1	51.8 ± 2.0
Fetal sex (m/f)	10/10	8/4
Placental weight (g)	667 ± 89	661 ± 100

* indicates *p* < 0.5, *** indicates *p* < 0.001.

## Supplementary Materials

Supplementary materials can be found at https://www.mdpi.com/1422-0067/21/3/834/s1.
