# Supplementary figures and images for: Maternal Overweight Downregulates MME (Neprilysin) in Feto-Placental Endothelial Cells and in Cord Blood
Source: Int J Mol Sci. 2020 Jan 28;21(3):834. doi: 10.3390/ijms21030834 (PMC7037888; doi:10.3390/ijms21030834)

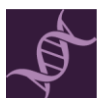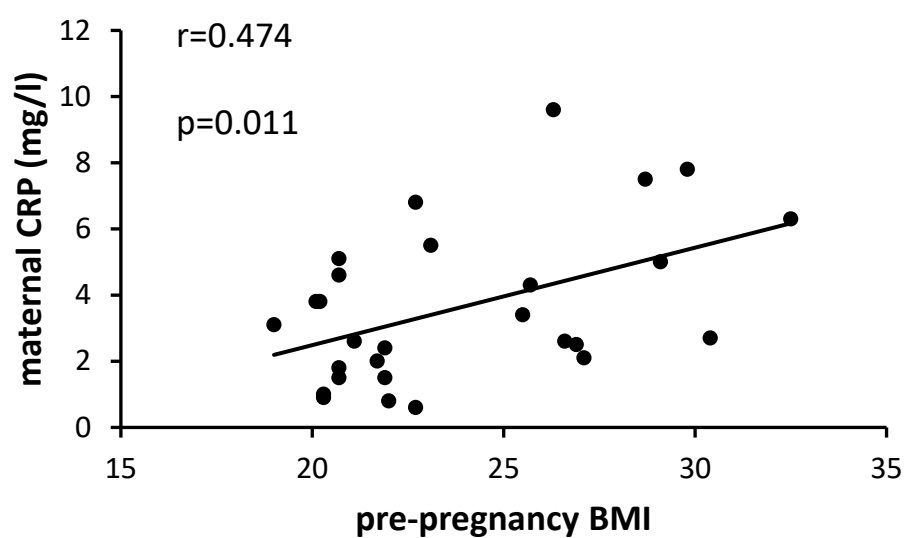

**Figure S1.** Maternal CRP at delivery correlates with maternal pre-pregnancy BMI (N=28).

Supplement: Supplementary file 1 [file ijms-21-00834-s001.pdf]
